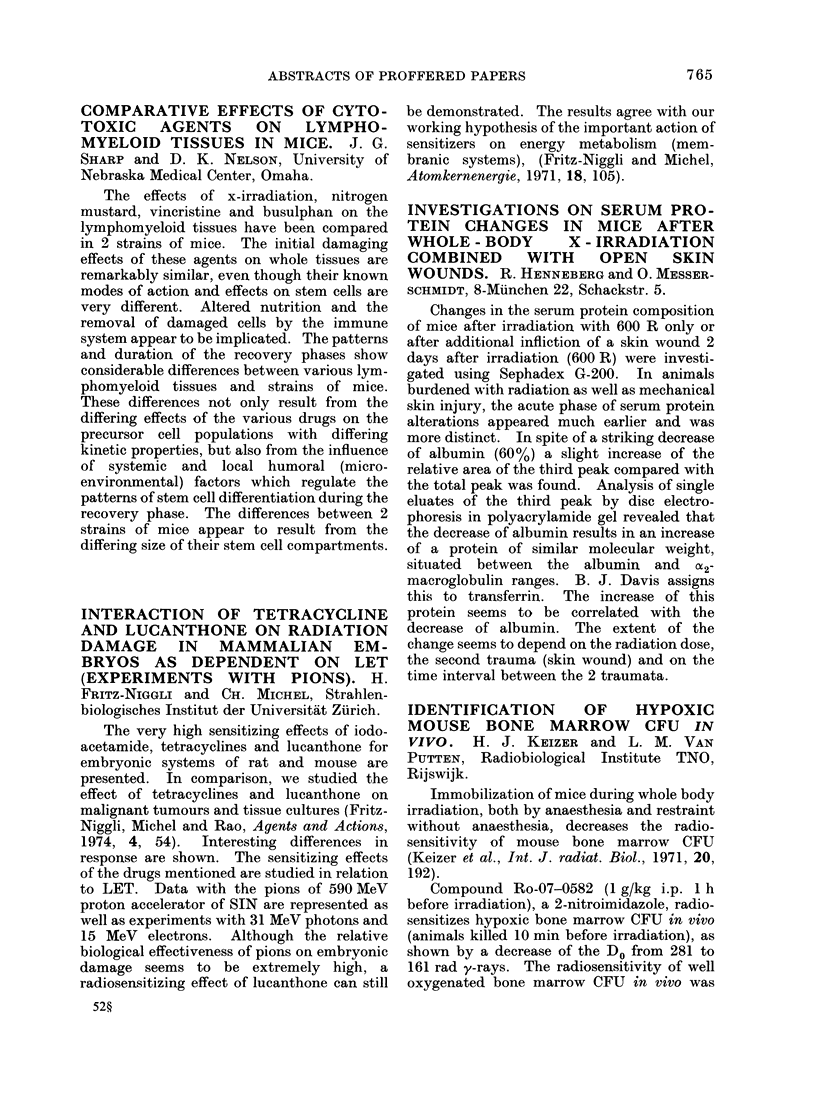# Proceedings: Ivestigations on serum protein changes in mice after whole-body x-irradiation combined with open skin wounds.

**DOI:** 10.1038/bjc.1975.336

**Published:** 1975-12

**Authors:** R. Henneberg, O. Messerschmidt


**INVESTIGATIONS ON SERUM PROTEIN CHANGES IN MICE AFTER WHOLE-BODY X-IRRADIATION COMBINED WITH OPEN SKIN WOUNDS.** R. HENNEBERG and O. MESSERSCHMIDT, 8-München 22, Schackstr. 5.

Changes in the serum protein composition of mice after irradiation with 600 R only or after additional infliction of a skin wound 2 days after irradiation (600 R) were investigated using Sephadex G-200. In animals burdened with radiation as well as mechanical skin injury, the acute phase of serum protein alterations appeared much earlier and was more distinct. In spite of a striking decrease of albumin (60%) a slight increase of the relative area of the third peak compared with the total peak was found. Analysis of single eluates of the third peak by disc electrophoresis in polyacrylamide gel revealed that the decrease of albumin results in an increase of a protein of similar molecular weight, situated between the albumin and $\alpha_2$-macroglobulin ranges. B. J. Davis assigns this to transferrin. The increase of this protein seems to be correlated with the decrease of albumin. The extent of the change seems to depend on the radiation dose, the second trauma (skin wound) and on the time interval between the 2 traumata.